# Avian leukosis virus subgroup J induces its receptor--chNHE1 up-regulation

**DOI:** 10.1186/s12985-016-0517-3

**Published:** 2016-04-02

**Authors:** Weiguo Feng, Wei Meng, Liming Cai, Xiyao Cui, Zhifang Pan, Guihua Wang, Ziqiang Cheng

**Affiliations:** College of Veterinary Medicine, Shandong Agricultural University, Tai’an, China; Weifang Medical University, Weifang, China; Shandong Provincial Key Laboratory of Animal Biotechnology and Disease Control and Prevention, Shandong Agricultural University, Tai’an, China

**Keywords:** Avian leukosis virus subgroup J (ALV-J), Chicken sodium hydrogen exchanger isoform 1 (chNHE1), Receptor, Up-regulation

## Abstract

**Background:**

Avian leukosis virus subgroup J (ALV-J) is an oncogenic retrovirus which causes immunosuppression and neoplasia in meat-type and egg-type chickens. ALV-J infects host cells via specific interaction between the viral Env and the cell surface receptor —chicken sodium hydrogen exchanger type 1 (chNHE1). NHE1 involved in altering the cellular pH and playing a critical role in tumorigenesis. However, little is known about the other relationship between ALV-J and chNHE1.

**Methods and results:**

In ALV-J infected DF-1 cells, the mRNA level of chNHE1 was up-regulated with time-dependent manner tested by real time PCR, and accordingly, intracellular pH was increased tested by spectrofluorometer. In vivo, the mRNA level of chNHE1 was determined by real time PCR in ALV-J infected experimental chickens and field cases. The result showed that the mRNA level of chNHE1 was up-regulated after virus shedding, especially in continuous viremic shedders (CS group). However, no significant difference was found between non-shedding group (NS group) and control group. In field cases, mRNA level of chNHE1 was positively correlated with increasing ALV-J load in tumor bearing and immune tolerance chickens. Furthermore, immunohistochemistry results showed that the protein expression of chNHE1 was up-regulated in different organs of both experimental chickens and tumor bearing chickens compared with the control.

**Conclusion:**

Taken together, we conclude that ALV-J induces chNHE1 up-regulation in viremia and neoplasia chickens.

## Background

Avian leukosis viruses (ALVs) are a group of avian oncogenic retroviruses which cause immunosuppression and tumors in host birds. Six subgroups (A–E and J) of ALV isolated from chicken are classified according to the virus neutralization, viral interference and the host range [1]. Subgroup J (ALV-J) was reported in 1991 and then broke out in meat-type and egg-type chickens during the 2000s [2–7]. ALV-J evolves with the sequence variations rapidly in the hypervariable regions (hr) of the envelope surface protein (SU) and 3′untranslated region (3′UTR) [3]. ALV-J mainly causes myeloid leukosis and various tumors in field or experimental infection [8]. The neoplastic and non-neoplastic diseases induced by ALV-J in both meat and egg type chickens represent a serious problem for the chicken industry now. ALV-J infect host cells via especial interactions between the viral envelope glycoprotein (Env) and the cell surface receptor, chicken sodium hydrogen exchanger isoform 1 (chNHE1), a functional receptor [9].

Sodium hydrogen exchanger type 1 (NHE1) is an H^+^ extruder, catalyzing the exchange of the intracellular H^+^ for the extracellular Na^+^ and regulating intracellular pH and cell volume [10]. Increased activity of NHE1 and resulting intracellular alkalinization are considered necessary for oncogenic transformation [11] and tumor development [12]. NHE1 activity is regulated by different intracellular and extracellular stimuli, such as acidosis, growth factors and oncogenes [13, 14]. In mammals, NHE1 is expressed abundantly in heart and playing an important role in cardiac failure and hypertrophy [15]. Moreover, ALV-J infection often leads to cardiomyopathy in chicken. These data mean a possible direct role of chNHE1 in myocardial injury during ALV-J infection.

However, little is known about the regulation of ALV-J on chNHE1 expression. In present study, we attempted to determine the relationship between ALV-J infection and chNHE1 expression.

## Results

### Transcription of chNHE1 and measurement of intracellular pH (pHi) in ALV-J infected DF-1 cells

**Fig. 1:**
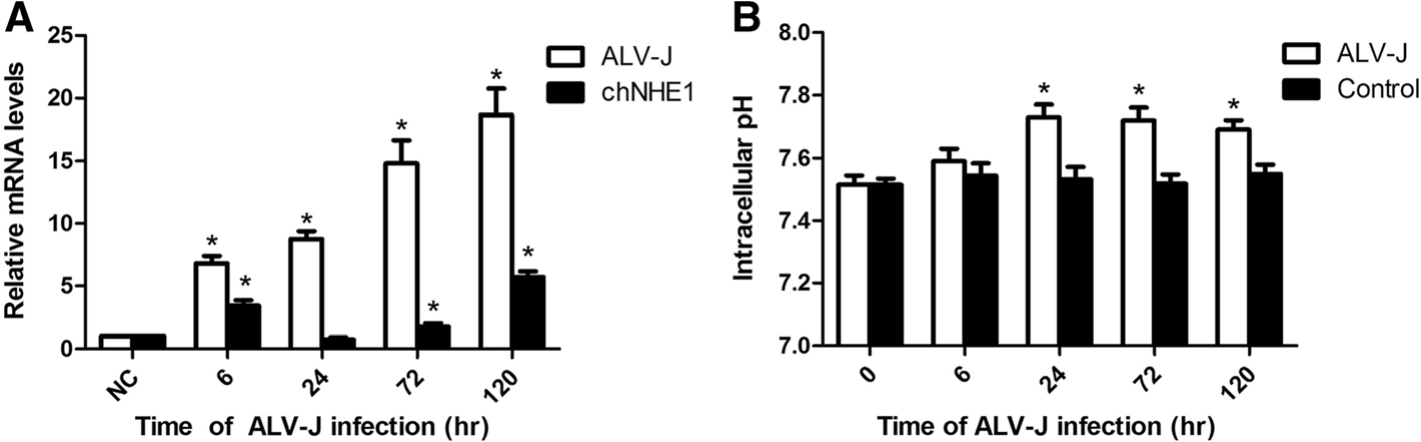
The mRNA level of chNHE1 and measurement of intracellular pH (pHi) in DF-1 cells at different time-points post infection. **a** DF-1 cells were infected by ALV-J with 10^3^ TCID_50_. The relative mRNA of ALV-J and chNHE1 were monitored by real-time RT-PCR at different times post infection. The results are representative of three independent experiments, and performed in triplicate. *P < 0.05 denotes a statistically significant difference comparing with control group. Error bars indicate SE. **b** DF-1 cells were infected by ALV-J at TCID_50_ of 10^3^. The intracellular pH was assessed by spectrofluorometer using the BCECF-AM at different times post infection. The results are representative of three independent experiments, and performed in triplicate. *P < 0.05 ALV-J infected cells versus control cells. Error bars indicate SE

To investigate whether ALV-J regulates chNHE1 expression and affects the pHi in vitro, we tested mRNA level of chNHE1 and pHi value in ALV-J infected DF-1 cells. As shown in Fig. 1a, the mRNA level of chNHE1 was up-regulated by ALV-J with wave-like curve, increasing at 6 h then decreasing at 24 h and increasing at 72 h again. The results indicated that ALV-J up-regulates the expression of chNHE1 with time-dependent manner. Furthermore, we tested the pHi of ALV-J infected DF-1 cells. In contrast to the control, the ALV-J infection led to rapid increase of pHi up to 7.7 at 24 h and maintain until 120 h (Fig. 1b). The results indicated that ALV-J up-regulates the expression of chNHE1 and then affects pHi.

### Infection status of experimental chickens

To further investigate the regulation of ALV-J on chNHE1, experimental infection of chickens was performed. After infection of ALV-J, nine chickens were hatched, and they all showed antibody negative for ALV-J. The result of p27 test was showed in Table 1. No 1, No 4 and No 5 chicken had not shed virus. No 2, No 3 and No 9 chicken begun shedding virus at day 17, day 19 and day 17, respectively. No 6, No 7 and No 8 continuously shed virus from day 1. Thus, the 9 chickens can be classified 3 types of shedder, non-shedders (V-A-, NS), late shedders (V + A-, LS), and continuous shedders (V + A-, CS). The kidneys of three types of shedders were tested for the mRNA level of ALV-J and chNHE1 by real time PCR.

**Table 1:** Result of p27 test in experimental infection of ALV-J

Day	1	2	3	4	5	6	7	8	9	NC
1	0.08	0	0	0	0	**1.48**	**1.11**	**1.25**	0	0
3	0.01	0.05	0.01	0.04	0.06	**1.31**	**1.08**	**1.14**	0.02	0.05
5	0.03	0.13	0.02	0.02	0.03	0.15	**0.41**	**0.48**	0.03	0.03
7	0.11	0.03	0	0	0	0.1	0.13	0.16	0.18	0
9	0	0.03	0	0	0	**0.71**	**1.68**	**1.16**	0	0
12	0	0.02	0	0	0	**0.5**	**0.34**	**0.28**	0.02	0
15	0	0	0	0	0	**1.11**	0.13	0.18	0	0
17	0	**0.72**	0.12	0	0	**0.38**	**1.06**	**0.89**	**0.21**	0
19	0	**0.96**	**0.48**	0	0.07	**2.27**	**1.33**	**1.23**	**0.65**	0

The values indicate shedding p27 antigen of ALV-J. The cut-off (s/p ratio) of 0.2 was recommended by the manufacturer. *NC* negative control

Bold data indicate that the value is more than 0.2

### The mRNA expression of ALV-J and chNHE1 in experimental chickens

**Fig. 2:**
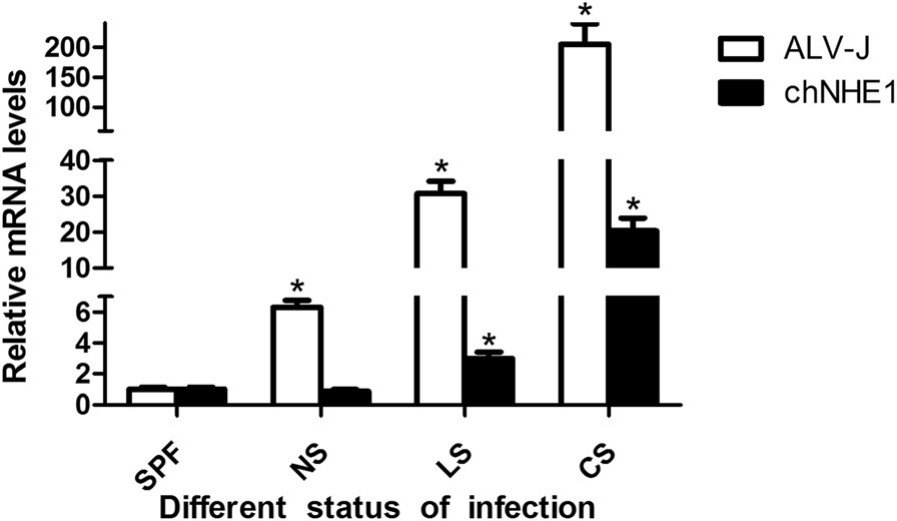
The mRNA level of ALV-J and chNHE1 in different infection status of experimental infection chickens. SPF group: negative control group; NS group: no shedding virus group; LS group: shedding virus at late stage of experimental period; CS group: continuous shedding virus from day 1. RNA was extracted from kidney, and then to detect mRNA level of ALV-J and chNHE1 by real time RT-PCR. Data are representative of the results of three independent experiments, both performed in triplicate. *P < 0.05 denotes a statistically significant difference comparing with control group. Error bars indicate SE

The birds from different status of infected birds (NS, LS and CS) were euthanatized at terminal day (19 days after hatching), and RNA extracted from kidney was used to test the mRNA level of ALV-J and chNHE1 by real time PCR. As shown in Fig. 2, the mRNA level of chNHE1 increased with ALV-J increasing. Moreover, different status of infection (NS, LS and CS) showed different mRNA level of ALV-J and chNHE1. In CS group (shedding from day 1), the mRNA level of ALV-J and chNHE1 showed an extremely significant increase when compared with LS, NS and control group (*P* < 0.05). In LS group (shedding from day 17), the mRNA level of ALV-J and chNHE1 was significant lower than CS group and higher than control group (*P* < 0.05). In NS group, no significant difference was found when compared with control group (*P* > 0.05). The results indicated that ALV-J up-regulates the mRNA level of chNHE1 associated with virus shedding, especially in continuous viremic shedders.

### The protein expression and distribution of chNHE1 in tissues of ALV-J infected chickens

**Fig. 3:**
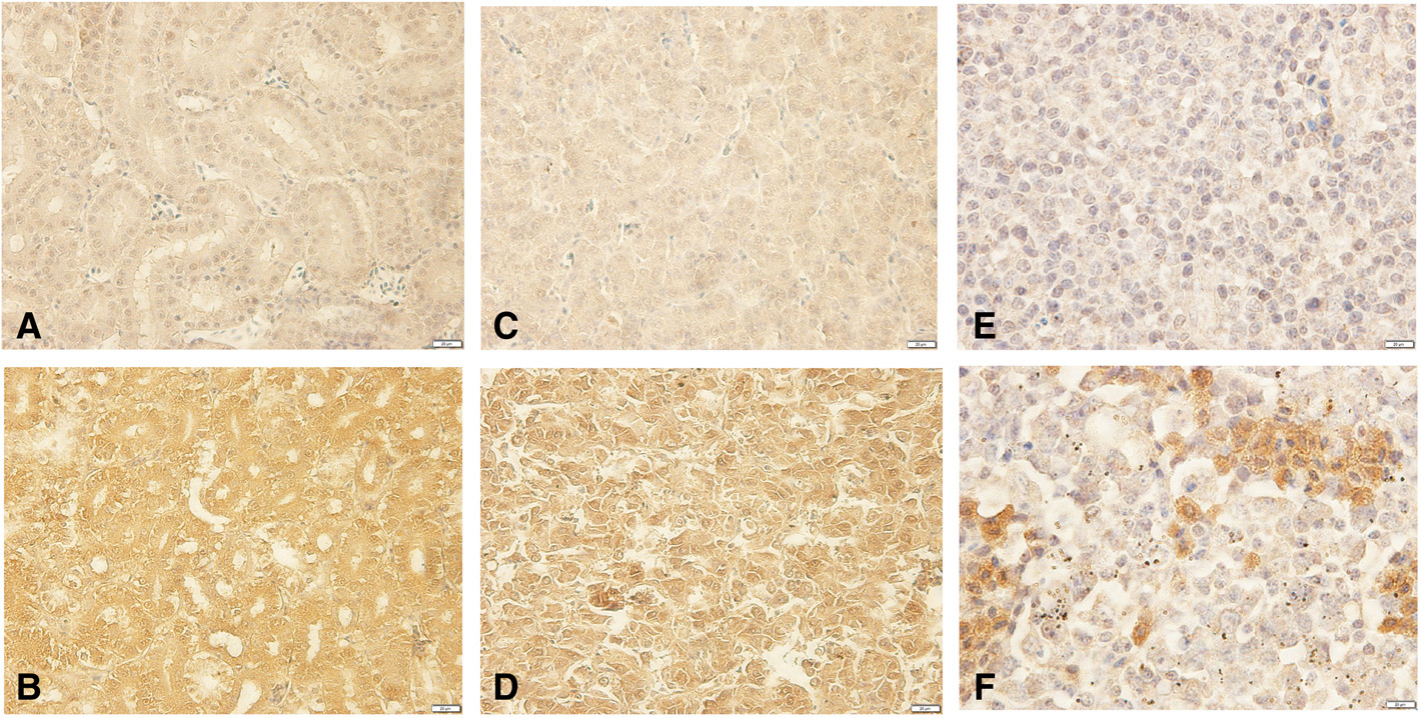
The protein expression and distribution of chNHE1 in tissues of ALV-J infected chickens. The brown staining in cell membrane indicated chNHE1 positive signals. **a** Control of kidney, IHC, 400×; **b** ALV-J infection of kidney, IHC, 400×; **c** Control of liver, IHC, 400×; **d** ALV-J infection of liver, IHC, 400×; **e** Control of bone marrow, IHC, 400×; **f** ALV-J infection of bone marrow; IHC, 400×. Control: uninfected chicken tissue

Next, we detected the protein expression and distribution of chNHE1 in tissues of ALV-J infected chickens by immunohistochemistry analysis using specific anti-chNHE1 antibody prepared by our lab. The brown staining in cell membrane indicated chNHE1 positive signals. The results showed that the staining intensities of chNHE1 in ALV-J infection group (CS group) were higher than the control group. The increased chNHE1 protein was present in renal tubular epithelial cell of kidney (Fig. 3a-b), hepatocytes of liver (Fig. 3c-d), myeloid cells of bone marrow (Fig. 3e-f), the epithelial cells of the adrenal gland, germinal epithelium cells of the testis, oocytes of primary follicle and growing follicle, some lymphocytes of the thymus and bursa of Fabricius, myocardial cells of the heart, epithelial cells and smooth muscle cells of the gastrointestinal tract (data not shown). No significant change of chNHE1 expression was observed in ALV-J infected brain (data not shown). The results indicated that ALV-J induces up-regulation of chNHE1 protein expression in most tissues.

### chNHE1 expression in ALV-J induced tumor bearing chickens from field cases

**Fig. 4:**
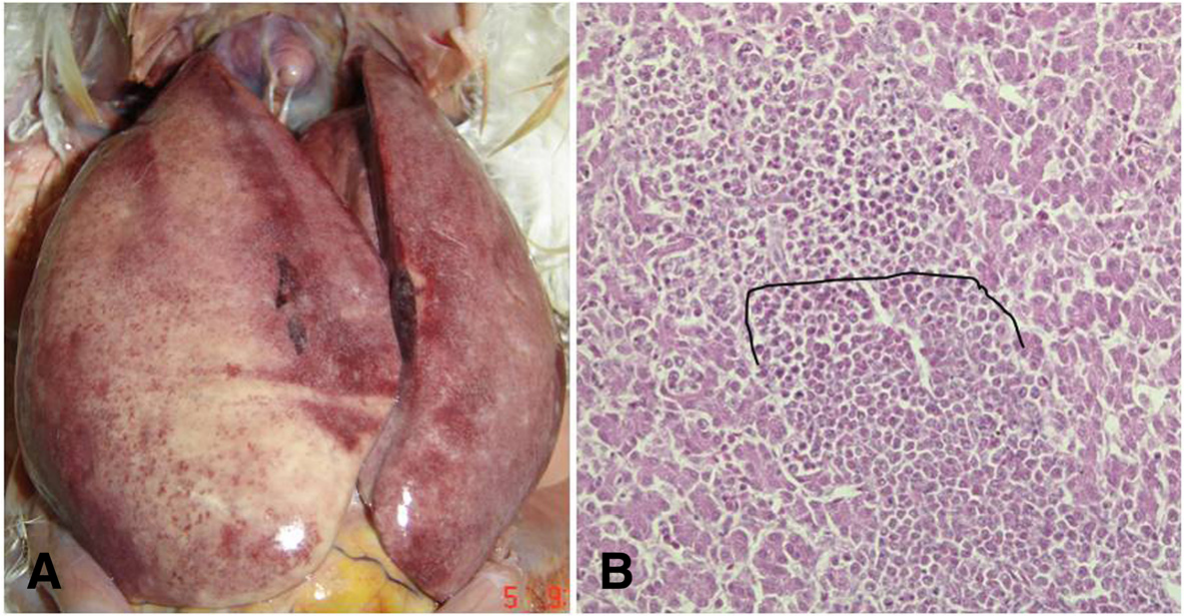
Myelocytoma and lymphoma induced by ALV-J in liver. **a** The gross lesion of myelocytoma and lymphoma liver; **b** The upper of black cure line is lymphoma, and the lower of black cure line is myelocytoma in liver, HE stain, 200×

**Fig. 5:**
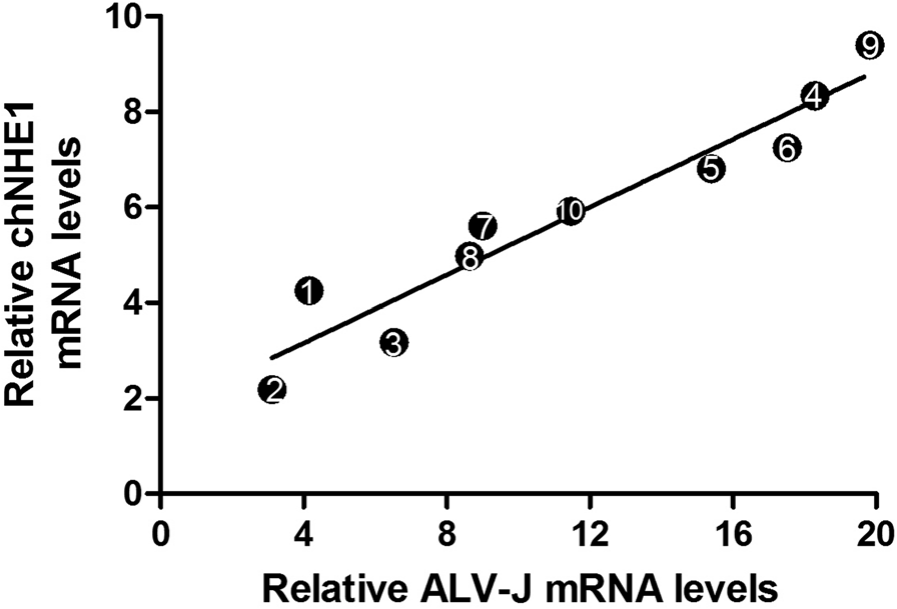
The relative quantity of mRNA of ALV-J and chNHE1 in ALV-J induced tumor bearing chickens. RNA was extracted from kidney, and then to detect mRNA level of ALV-J and chNHE1 by real time RT-PCR. Data are representative of the results of three independent experiments, both performed in triplicate. Pearson Bivariate Correlations test was used to determine the correlation between the mRNA level of chNHE1 and ALV-J. The date points show the number of the chickens

**Fig. 6:**
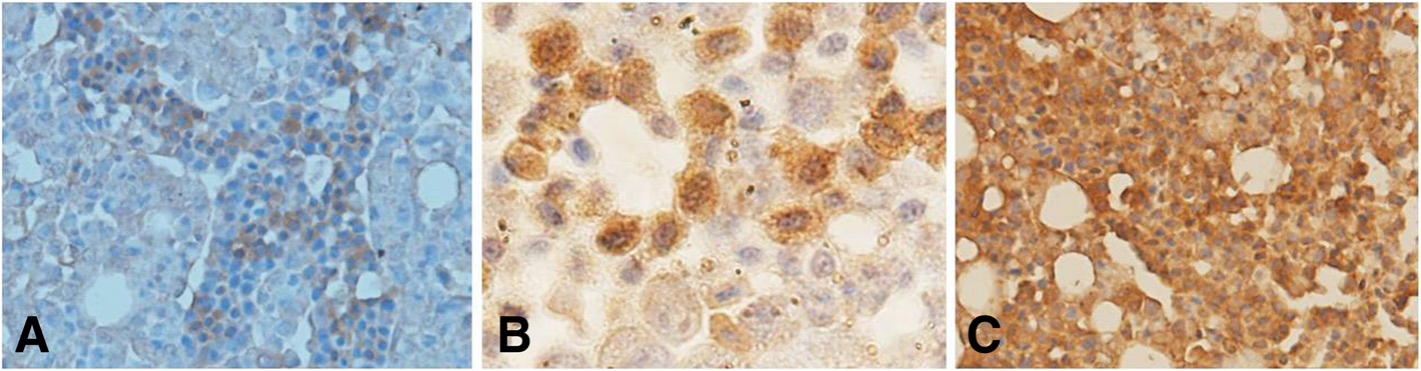
The protein expression of chNHE1 in ALV-J induced tumor bearing bone marrow. The brown staining indicated chNHE1 positive signals. **a** The slight stained in normal bone marrow, IHC, 400×; **b** Some myeloid cells were strongly stained in ALV-J infected bone marrow (no tumor present), IHC, 1000×; **c** All cells were strongly stained in myelocytoma in bone marrow, IHC, 200×

All ten field layer chickens (commercial layer chickens) showed antibody negative for ALV-J and p27 positive (indicating the presence of antigen of ALV-J). Different types of tumor were observed in these field chickens. Myelocytoma was the predominant type of tumor induced by ALV-J in the ten chickens, sometimes companied with lymphomas (Fig. 4) or other tumors, such as hemangioma, erythroblastosis and fibrosarcomas (Tumor spectrum was showed in Table 2). The mRNA of ALV-J and chNHE1 in kidneys was assessed by real time PCR. As shown in Fig. 5, the mRNA level of chNHE1 increased with ALV-J load increasing in these field case (*r*^2^ = 0.91, *P* < 0.05). Furthermore, the immunohistochemistry analysis showed that the staining intensities of chNHE1 in ALV-J induced tumor bearing bone marrow was significantly higher than ALV-J infected (no tumor present) bone marrow and normal bone marrow group (Fig. 6). All of these suggested that there is close relationship between ALV-J and chNHE1 in tumor bearing chickens.

**Table 2:** Field isolates of ALV-J and induced tumor spectrum

Isolate no	Tumor spectrum and distribution
1	ML (kidney, liver, spleen, lung), HG (claw)
2	LL (liver, lung)
3	ML (kidney, liver, spleen), LL (liver), FS (pectoralis)
4	ML (kidney, liver), LL (liver), HG (lung)
5	ML (liver), EB (kidney, liver,)
6	ML (liver, spleen, intestine),LL (liver, spleen, lung), AN (pancreas, intestine)
7	HG (intestine, pancreas)
8	ML(kidney)
9	ML (kidney, bone, liver, spleen, lung, pancreas, intestine), EB (liver, spleen), LL (heart)
10	HG (intestine, pancreas)

*ML* myelocytomas, *HG* haemangiomas, *EB* erythroblastosis, *LL* lymphocytomas, *FS* fibrosarcoma, *AN* aneurysm

## Discussion

Understanding the viral–receptor interactions and the cellular pathways regulated by this interaction are necessary for development of new methods to control virus infection. Ning Chai demonstrated that chNHE1 was the receptor of ALV-J [9]. NHE1 is an H^+^ extruder, catalyzing the exchange of the intracellular H^+^ for the extracellular Na^+^, regulating pHi and resulting in tumorigenesis [10–12]. Meanwhile, ALV-J could cause neoplasia in meat and egg type chickens. Thus, we speculated that chNHE1 is a key factor in virus cell entry and tumor formation through regulation of chNHE1 in ALV-J infection. In this paper, we attempt to know if ALV-J can regulate chNHE1.

In DF-1 cells, the transcript of chNHE1 was up-regulated by ALV-J with time-dependent manner, and increased the pHi up to 7.7. It is well known that the pHi of normal cells is about 6.9-7.4 [16]. The activation of NHE1 function drive the cytoplasmic alkalization leading the pHi increased to 7.2-7.8 which can inhibit cell apoptosis and promote cell proliferation, leading to tumorigenesis [17]. It suggested that chNHE1 is up-regulated by ALV-J and may be crucial for tumor formation. In order to confirm the up-regulation of chNHE1 induced by ALV-J, experimental infection of ALV-J was designed.

In experimental infection, the mRNA level of chNHE1 was also elevated by ALV-J. The mRNA level of chNHE1 was up-regulated significantly in LS and CS group which indicated that ALV-J induces up-regulation of mRNA level of chNHE1 associated with virus shedding, especially in persistent viremic shedders. Furthermore, our results suggested a possibility that ALV-J and chNHE1 activate each other. In this mode, ALV-J up-regulates the chNHE1, while the up-regulation of chNHE1 promoted the replication and release of ALV-J.

Protein are executor of function, therefore, we investigated the regulation of ALV-J on chNHE1 protein expression by immunohistochemistry analysis. The protein level expression of chNHE1 was increased in most tissues during ALV-J infection. It is well known that high expression of NHE1 is necessary for tumor formation [12]. Elevated NHE1 regulate cell cycle/proliferation, cell migration/invasion and cell death by activation of intracellular downstream signaling pathways resulting in tumorigenesis [18]. Hence, we speculated that tumor formation induced by ALV-J is through up-regulation of chNHE1. This could explain why tumors induced easily in kidney, liver and bone marrow in ALV-J infection. Unfortunately, there is no commercial antibody of chNHE1, as well as the antibody prepared by our lab is suitable for immunohistochemistry rather than western blot due to designing for space configuration of the epitope. As a result, western blot was not carried out to detect expression of chNHE1.

Finally, the regulation of ALV-J on chNHE1 was confirmed in ALV-J induced tumor bearing chickens. We found that the mRNA level of chNHE1 was positive correlation with ALV-J load in immunological tolerance and tumor bearing shedder chickens (*r*^2^ = 0.91, *P* < 0.05). Meanwhile, the protein level of chNHE1 in tumorous bone marrow was significantly higher than ALV-J infected (no tumor present) bone marrow and normal bone marrow. The results strongly suggested that up-regulation of chNHE1 by ALV-J play a key role in tumor formation, however, the signal pathway or the mechanism need to be further studied.

## Conclusions

In summary, we demonstrated that ALV-J induces chNHE1 up-regulation in viremia and neoplasia chickens. The results indicate that chNHE1 plays a pivotal role in ALV-J infection and tumorigenesis, and offer a targeted strategy to control of ALV-J by regulating chNHE1.

## Methods

### Ethical approval

This study was carried out in strict adherence to the recommendations in the Guide for the Care and Use of Laboratory Animals of the National Institutes of Healthy. The protocol was approved by the Committee on the Ethics of Animal of Shandong (Permit Number: 20100326).

### Cell culture and virus infection

DF-1 cells were cultured in 25 cm^2^ flasks (Corning, Shanghai, China) in Dulbecco’s modified Eagle’s medium (DMEM; Invitrogen, Shanghai, China) supplemented with 10 % fetal bovine serum (FBS; Gibco, Shanghai, China) plus 100 *μ*g/m*l* of penicillin and streptomycin. The cultures were incubated at 37 °C and 5 % CO_2_.

DF-1 cells were seed in 25 cm^2^ flasks in 10 % FBS DMEM and infected by ALV-J (strain of NX0101) at 50 % tissue culture infective dose (TCID_50_) of 10^3^ until cells grew about 90 % confluence, and the DF-1 cells were maintained in DMEM supplemented 1 % FBS in 37°C and 5 % CO_2_ after infection. The DF-1 cells were harvested at 6 h, 24 h, 72 h and 120 h of post-infection.

### Measurement of pHi

The pHi of DF-1 cells was measured by spectrofluorometer using 2′, 7′-bis-(2-carboxyethyl)-5 (6)-carboxyfluorescein, acetoxymethylester (BCECF-AM; Beyotime, Shanghai, China) which is a pH-sensitive fluorescent probe [19]. The cell suspensions were washed in phosphate buffer saline (PBS) and labeled with BCECF-AM. The excitation wavelength is 488 nm, and the emission wavelength is 530 nm. A standard curve was done with nigericin in every experiment to calibrate pHi of DF-1 cells as previous study [19].

### Egg inoculation and incubation

Fertile eggs were purchased from special pathogens free (SPF) broiler breeders (Saishi Co.Ltd, Jinan, China). Twenty eggs were divided into two equal groups at day 6 of incubation and were yolk sac inoculated. Each embryo of group 1 was injected 100 *μl* with 10^4^ TCID_50_ of NX0101 strain, and embryos of group 2 was used as control group. The eggs were incubated and hatched separately per experimental group. The incubation and hatch temperature was 37 °C. After hatching, cloaca swabs and blood were taken starting at day 1 and was then performed at interval 1 day until the experimental terminal (19 days). Cloaca swabs were tested for group specific antigen (p27), and sera were tested for antibody of ALV-J. The birds were euthanatized by cervical dislocation and necropsied at terminal day, and RNA extracted from kidney was used to test relative amount of ALV-J and chNHE1 by real time PCR.

### Antibody and p27 test

Antibody against ALV -J was detected by analysis of serum samples with enzyme-linked immunosorbent assay (ELISA) kits (IDEXX Laboratories, Westbrook, U.S.A.). The presence p27 was determined on cloaca swabs using a commercial antigen capture ELISA kit (IDEXX Laboratories, Westbrook, U.S.A.).

### Histopathology observation

At postmortem, samples of heart, kidney, liver, proventriculus, duodenum, pancreas, lung, skeletal muscle, spleen, thymus, bursa of Fabricius, ovary, adrenal gland, brain, bone marrow from field cases and experimental groups were examined histologically. Tissues were fixed in 10 % neutral buffered formalin. The tissues were embedded in paraffin, sectioned about 4 *μ*m thickness, and stained with hematoxylin and eosin (H&E) for observation.

### Real time PCR

The mRNA levels of ALV-J and chNHE1 in DF-1 cells and kidney were quantitated by real time PCR. The primers were showed in Table 3. Real time PCR was performed using the SYBR Premix Ex Taq Kit (ROCHE Molecular Biochemicals, Indianapolis, U.S.A.). The final reaction volume is 12.5 *μl* containing 0.5 *μl* of ROX Reference Dye (50×), 0.5 *μl* of each primer (10 mM), 2 *μl* of cDNA, and 9 *μl* water. The reaction was conducted in a real time PCR as follows: 95 °C for 30 s followed by 43 cycles of denature at 95 °C for 5 s, annealing at 60 °C for 34 s, and extension at 72 °C for 13 s. The melt curve was checked to verify that the products were specific.

**Table 3:** Primers used in real time PCR

Gene	Primer	Primer sequence	Size of PCR product
gp85	Pf	GTCACTAATGTTACTGCTTGCG	434 bp
	Pr	GAACTACACCAATTAGAAAG	
chNHE1	Pf	CTGAACAAACAGCACCACAAC	291 bp
	Pr	GAGGAGGAAGAGGAAGAAGATG	
GAPDH	Pf	GGTGGTGCTAAGCGTGTTA	179 bp
	Pr	CCCTCCACAATGCCAA	

The gp85 primers were used for detecting the mRNA level expression of ALV-J

Assays were performed in triplicate and average threshold cycle (CT) values were used to determine relative concentration differences based on the ∆∆CT method [20]. For all samples including the negative control, the difference between the CT of the target gene (ALV-J or chNHE1) and internal control (∆CT = CT target gene - CT internal control) was calculated. And then the difference between the ∆CT of experimental sample and negative control (∆∆CT = (CT target gene - CT internal control) experimental sample - (CT target gene - CT internal control) negative control) was calculated. The expression level of target gene was calculated by 2^-∆∆CT^.

### Immunohistochemistry

The kidney, liver and bone marrow from CS group (V + A-, continuous viremic shedders) and tumor tissue were fixed with 10 % formalin, paraffin-embedded and sectioned in 4 *μ*m. Immunohistochemistry was performed as previous study [21]. Briefly, sections were pre-treated using 3 % H_2_O_2_ for 20 min, blocked using 10 % bovine serum albumin for 20 min. Slides were incubated with primary antibody (mouse anti-chNHE1 prepared by our lab) at a dilution of 1:1000 overnight at 4 °C. After washing with PBS, slides incubated with secondary antibody (anti-mouse IgG, Santa Cruz, CA, USA) at a dilution of 1:1000 for 1 h at room temperature. After washing with PBS, coloration was performed by 3, 3-diaminobenzidine (DAB) and counterstained using hematoxylin. Finally, slides were observed under the light microscopy. (Negative control: primary antibody was replaced by rabbit IgG, data not shown).

### Field cases of ALV-J single infection

Ten field cases of ALV-J single infection were identified by histopathology, PCR and immunofluorescence assay (IFA) from 84 suspected ALV-J infection Hy-line layer chickens. Due to kidney is high associate with viral shedding, it was chose to extract RNA, and then to detect mRNA level of ALV-J and chNHE1 by real time PCR.

### Statistical analysis

There were at least three replicates for each treatment. In the experimental infection, statistical comparisons were made by using an independent sample *t*-test. In field cases, a Pearson Bivariate Correlations test was used to determine the correlation between the mRNA level of chNHE1 and ALV-J load. The soft ware used was SPSS (Statistics Package for Social Science).
